# Association of self-reported sports volume and discipline with atrial arrhythmia prevalence in middle-aged males

**DOI:** 10.1093/ehjopen/oeag089

**Published:** 2026-05-22

**Authors:** Jarne De Paepe, Astrid Witters, Ruben De Bosscher, Christophe Dausin, Rik Pauwels, Boris Delpire, Youri Bekhuis, Hein Heidbuchel, Guido Claessen, Andre La Gerche, Rik Willems, Tomas Robyns

**Affiliations:** Department of Cardiovascular Sciences, KU Leuven, Herestraat 49, Leuven B-3000, Belgium; Department of Cardiology, University Hospitals Leuven, Herestraat 49, Leuven B-3000, Belgium; Department of Cardiovascular Sciences, KU Leuven, Herestraat 49, Leuven B-3000, Belgium; Department of Cardiovascular Sciences, KU Leuven, Herestraat 49, Leuven B-3000, Belgium; Department of Cardiology, University Hospitals Leuven, Herestraat 49, Leuven B-3000, Belgium; Department of Movement Sciences, KU Leuven, Tervuursevest 101, Leuven B-3001, Belgium; Department of Cardiovascular Sciences, KU Leuven, Herestraat 49, Leuven B-3000, Belgium; Department of Cardiology, University Hospitals Leuven, Herestraat 49, Leuven B-3000, Belgium; Department of Cardiology, Hartcentrum, Jessa Ziekenhuis, Stadsomvaart 11, Hasselt 3500, Belgium; Faculty of Medicine and Life Sciences, Cardiology and Organ Systems, LCRC, Hasselt University, Martelarenlaan 42, Hasselt 3500, Belgium; Department of Cardiovascular Sciences, KU Leuven, Herestraat 49, Leuven B-3000, Belgium; Department of Cardiology, University Hospitals Leuven, Herestraat 49, Leuven B-3000, Belgium; Department of Cardiology, Hartcentrum, Jessa Ziekenhuis, Stadsomvaart 11, Hasselt 3500, Belgium; Faculty of Medicine and Life Sciences, Cardiology and Organ Systems, LCRC, Hasselt University, Martelarenlaan 42, Hasselt 3500, Belgium; Department of Cardiovascular Sciences, KU Leuven, Herestraat 49, Leuven B-3000, Belgium; Department of Cardiology, University Hospitals Leuven, Herestraat 49, Leuven B-3000, Belgium; Department of Cardiovascular Sciences, GENCOR, University of Antwerp, Universiteitsplein 1, Antwerp 2610, Belgium; Department of Cardiology, University Hospital Antwerp, Drie Eikenstraat 655, Edegem 2650, Belgium; Department of Cardiology, Hartcentrum, Jessa Ziekenhuis, Stadsomvaart 11, Hasselt 3500, Belgium; Faculty of Medicine and Life Sciences, Cardiology and Organ Systems, LCRC, Hasselt University, Martelarenlaan 42, Hasselt 3500, Belgium; Heart, Exercise & Research Trials (HEART) Lab, St Vincent’s Institute, Princes street 9, Melbourne, VIC 3065, Australia; Department of Medicine, The University of Melbourne, Parkville, Melbourne, VIC 3010, Australia; Victor Chang Cardiovascular Research Institute, 405 Liverpool Street, Darlinghurst, NSW 2010, Australia; Department of Cardiovascular Sciences, KU Leuven, Herestraat 49, Leuven B-3000, Belgium; Department of Cardiology, University Hospitals Leuven, Herestraat 49, Leuven B-3000, Belgium; Department of Cardiovascular Sciences, KU Leuven, Herestraat 49, Leuven B-3000, Belgium; Department of Cardiology, University Hospitals Leuven, Herestraat 49, Leuven B-3000, Belgium

**Keywords:** Athlete’s heart, Endurance exercise, Training load, Atrial fibrillation, Self-reported

## Abstract

**Aims:**

A J-shaped relationship between exercise and atrial fibrillation (AF) or atrial flutter (AFL) has been described, but the influence of specific sport disciplines is unknown.

**Purpose:**

To examine the relationship between endurance exercise and AF/AFL prevalence in middle-aged men.

**Methods and results:**

We analysed questionnaire data from 3939 candidates for the Master@Heart study, assessing self-reported exercise history, cardiovascular risk factors and history of AF/AFL. Participants were divided into quartiles of lifetime endurance exercise hours: Q1 ≤ 1778, Q2 1779–5977, Q3 5978–12231, and Q4 > 12 231 h. Multivariable logistic regression was performed to assess the relationship between exercise volume and type and AF/AFL. AF/AFL prevalence was 7.5% and increased significantly across lifetime exercise quartiles (4.8%, 7.6%, 8.0%, and 9.6% from Q1 to Q4; *P* < 0.001). Adjusting for age, height, and traditional risk factors, Q2—Q4 had significantly higher odds ratios of AF/AFL compared to Q1, with the largest effect for Q4 (OR 2.16, 95% CI [1.48–3.15], *P* < 0.001). As sport, only cycling was independently associated with AF/AFL (OR 1.51 [1.11–2.07], *P* = 0.010).

**Conclusion:**

In middle-aged males, greater lifetime endurance exercise volume (>1778 h) was associated with progressively increased odds ratios of AF/AFL, with the greatest risk in the highest quartile (>12 231 h), independent of traditional risk factors. Cycling specifically was associated with AF/AFL. These findings support existing evidence that high-volume endurance exercise may increase AF/AFL risk, even in traditionally low-risk individuals, and furthermore suggest a possible sports-specific effect.

## Graphical Abstract

**Graphical Abstract:**
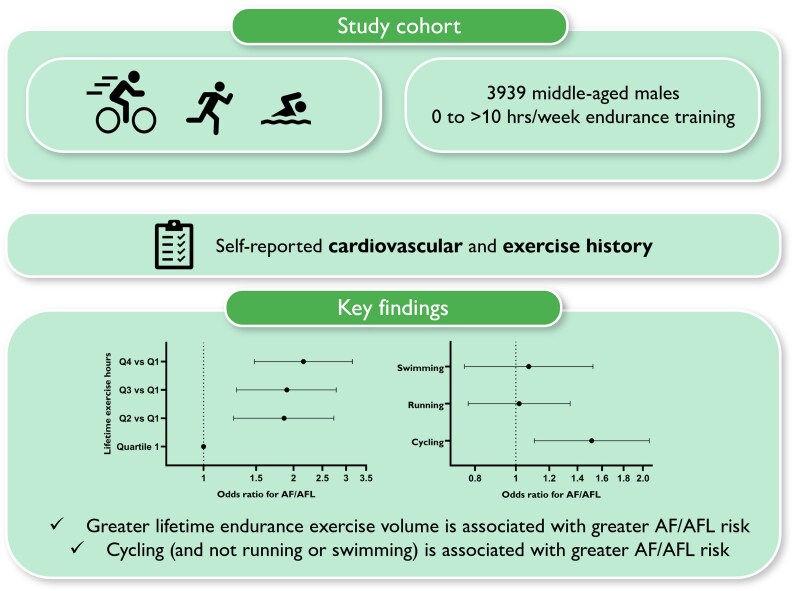

## Introduction

While the overall health benefits of regular endurance exercise are well established, emerging evidence suggests a potential association between high volume, high intensity endurance training and an increased risk of atrial fibrillation (AF). This relationship appears to be complex and non-linear: Moderate levels of physical activity are associated with a reduced risk of AF, whereas very high volumes of strenuous endurance exercise may confer an elevated risk,^1–4^ giving rise to the hypothesis of a J-shaped relationship between exercise volume and AF incidence. Although limited, evidence suggests a similar association for atrial flutter (AFL).^5^

The mechanisms underlying this relationship remain incompletely understood. It is plausible that moderate exercise reduces AF/AFL risk by favourably impacting traditional risk factors, including blood pressure, body weight, and glycaemic control.^6^ Conversely, high training loads may promote AF/AFL through mechanisms including biatrial structural remodelling, alterations in autonomic balance, and systemic inflammation.^7–10^

The hypothetical J-shaped relationship between endurance exercise and AF/AFL risk remains poorly defined at the level of the individual athlete. It is unclear to what extent the inflection point of the association curve is modified by exercise volume, sports discipline, and traditional cardiovascular risk factors. Adequately powered studies across different levels and disciplines of endurance sport are needed to guide future recommendations for athletes.

Our current analysis aims to evaluate the association between endurance exercise and AF/AFL history in a large cohort of middle-aged men using prospectively collected self-reported data.

## Methods

### Study population

This study presents a cross-sectional analysis of questionnaire data collected during the screening stage of the Master@Heart study. A detailed study design of Master@Heart has been published previously.^11^ Recruitment took place between October 2018 and May 2022 through a large-scale campaign using traditional media (e.g. television broadcasts) as well as social media platforms. Recruitment materials included content referring to the effects of endurance exercise on the heart, as well as materials specifically targeting non-athletic individuals, without reference to sports participation (i.e. healthy, non-smoking, non-obese men aged >45 years). The final inclusion and exclusion criteria were not disclosed at this stage. In all materials, the study purpose was presented as focusing on coronary artery disease. Individuals interested in participating were directed to a dedicated web portal (www.masteratheart.be), where they completed a predefined online screening questionnaire. The study protocol was approved by the local Ethics Committee (trial registration number S61336) and all participants provided written informed consent.

### Data collection

Participants completed an online questionnaire (www.masteratheart.be) that collected demographic data (gender, age, weight, body height), cardiovascular risk factors (smoking status, medication use, known cardiovascular conditions), and detailed exercise history. The complete questionnaire is provided in Supplementary File  *S1*.

### Endurance exercise definition

Participants were asked whether they engaged in weekly sports activities and, if so, to report the average number of hours per week per sport: a predefined list of 22 options (see Supplementary material) was provided, including both endurance and non-endurance disciplines. For duathlon and triathlon, hours for each component (running, cycling, swimming) were recorded separately.

Endurance sports were defined as cycling, running, swimming, rowing, duathlon, and triathlon. Total weekly hours of endurance exercise were calculated by summing the reported hours across all these sports. Lifetime exercise hours were calculated by multiplying reported weekly hours by the number of years of participation. Total lifetime endurance exercise exposure was then obtained by summing the lifetime hours across cycling, running, swimming and rowing. Participants were categorized into equal quartiles based on lifetime exercise hours. Quartile 1 included ≤1778 h; Quartile 2 included >1778 to ≤5977 h; Quartile 3 included >5977 to ≤12 231 h; and Quartile 4 included >12 231 h. Participants were considered active in a given sport if they reported ≥1 h per week.

### Outcome definition

The primary outcome was self-reported history of AF and/or AFL, as indicated on the questionnaire.

### Statistical analysis

Normality was tested using the Shapiro–Wilk test. Continuous variables are presented as mean (± standard deviation) or as median [25th–75th percentiles] and categorical variables as number (percentage). Between-group differences in continuous variables were assessed using independent *t*-test/ANOVA or Mann–Whitney U/Kruskal–Wallis test as appropriate. Dichotomous variables were compared using a chi-squared or Fisher’s exact test as appropriate.

Multivariable logistic regression models were constructed to evaluate the relationship of exercise volume and sports discipline with AF/AFL. Covariates were selected *a priori* based on clinical relevance and included age, body height, weight, smoking status, alcohol consumption, coronary artery disease, and use of antihypertensive, lipid-lowering, and antidiabetic medications. All covariates were entered simultaneously using the Enter method. Collinearity statistics were performed and found adequate at a VIF < 5. For the exercise volume model, the bottom quartile was chosen as the reference group. An additional logistic regression model was constructed including lifetime exercise hours as a continuous variable. To explore potential non-linear relationships, penalized smoothing splines with REML were applied to minimize overfitting. A sensitivity analysis excluding participants with weekly exercise volumes >20 h/week was conducted to rule out potential skewing of results by outliers.

A two-tailed *P*-value <0.05 was considered statistically significant. Data was analysed using SPSS Statistics version 29 (IBM Corporation, Armonk, NY, USA).

## Results

### Baseline characteristics by AF/AFL history

Of the 3939 completed questionnaires, 296 participants (7.5%) reported a history of AF/AFL. Participants with AF/AFL were significantly older than the group without AF/AFL (59 [53–65] vs. 55 [49–61] years, *P* < 0.001) and were slightly heavier (78 [73–85] vs. 77 [72–84] kg, *P* = 0.029). The AF/AFL group included more current or past smokers than the non-AF/AFL group (42% vs. 35%, *P* = 0.008) and also more frequently reported known coronary artery disease (11% vs. 4%, *P* < 0.001).

Use of medication for diabetes (4% vs. 1%, *P* = 0.002), hypertension (16% vs. 9%, *P* < 0.001) and dyslipidaemia (27% vs. 13%, *P* < 0.001) was more common among those with AF/AFL. Weekly endurance exercise volume was numerically higher in the AF/AFL group (8 [4–11] vs. 6 [3–10] h/week), though the difference did not reach statistical significance (*P* = 0.076). The AF/AFL group accumulated more lifetime exercise hours (7318 [3694–15 653] vs. 5809 [1666–12 061] h, *P* < 0.001).

No significant differences were observed in reported height or alcohol consumption between groups.

Sport type distribution also differed between groups: participants with AF/AFL were more likely to be cyclists (72% vs. 62%, *P* < 0.001) and less likely to be runners (40% vs. 47%, *P* = 0.013), while the proportion of swimmers was comparable (16% in both groups, *P* = 0.943). Due to the small number of rowers, separate analysis of this subgroup was not feasible. Finally, 12% of participants with AF/AFL reported no regular endurance training, compared to 14% of those without AF/AFL (*P* = 0.253).

Baseline population characteristics by AF/AFL history are summarized in *Table 1*.

**Table 1:** Baseline characteristics by AF/AFL history

	History of AF/AFL (*n* = 296)	No history of AF/AFL (*n* = 3643)	*P* value
Age (years)	59 [53–65]	**55 [49–61]**	**<0**.**001**
Body height (cm)	179 [175–183]	178 [174–183]	0.063
Weight (kg)	78 [73–85]	77 [72–84]	**0**.**029**
Alcohol consumption (units per week)	5 [3–8]	5 [2–8]	0.067
Current or past smoker—*n* (%)	125 (42)	1261 (35)	**0**.**008**
Coronary artery disease—*n* (%)	32 (11)	139 (4)	**<0**.**001**
Drugs			
Antidiabetic—*n* (%)	11 (4)	45 (1)	**0**.**002**
Antihypertensive—*n* (%)	46 (16)	328 (9)	**<0**.**001**
Lipid-lowering—*n* (%)	79 (27)	488 (13)	**<0**.**001**
Endurance exercise			
Lifetime exercise (hrs)	7318 [3694–15 653]	5809 [1666–12 061]	**<0**.**001**
Weekly exercise (hrs)	8 [4–11]	6 [3–10]	0.076
Cycling—*n* (%)	212 (72)	2244 (62)	**<0**.**001**
Running—*n* (%)	118 (40)	1726 (47)	**0**.**013**
Swimming—*n* (%)	48 (16)	585 (16)	0.943
Rowing—*n* (%)	1 (0)	32 (1)	0.511
No Regular Endurance Exercise—*n* (%)	34 (12)	505 (14)	0.253

Data presented as mean ± standard deviation, median [25th percentile—75th percentile] or number (%). Abbreviations—AF: atrial fibrillation, AFL: atrial flutter

### Baseline characteristics by lifetime exercise volume

Participants were divided into quartiles according to total lifetime endurance exercise hours: Q1 (*n* = 985), Q2 (*n* = 985), Q3 (*n* = 984), and Q4 (*n* = 985).

Participants in the highest quartile were significantly older than those in each of the other quartiles. Weight decreased progressively from Q1 to Q4, with the highest quartile having the lowest mean weight. Height did not differ significantly between groups. The proportion of current or past smokers also decreased progressively across quartiles of lifetime exercise hours, with Q4 having the lowest prevalence. Weekly alcohol consumption was lower in Q4, though pairwise comparisons were non-significant. The use of antidiabetic, antihypertensive and lipid-lowering medication was similar across quartiles.

Sports type varied significantly across exercise volume quartiles. Both lifetime and weekly exercise hours increased with each quartile. The proportion of cyclists increased progressively from Q1 to Q4, with 93% of participants in Q4 reporting cycling. A similar increasing trend across quartiles was observed for swimming, with 25% of Q4 reporting swimming. In contrast, running participation peaked in Q2 and Q3 (57% in both quartiles) and was lower in Q1 and Q4. Rowing participation was minimal and did not differ significantly. 55% of participants in Q1 did not engage in endurance exercise on a regular basis.

**Figure 1:**
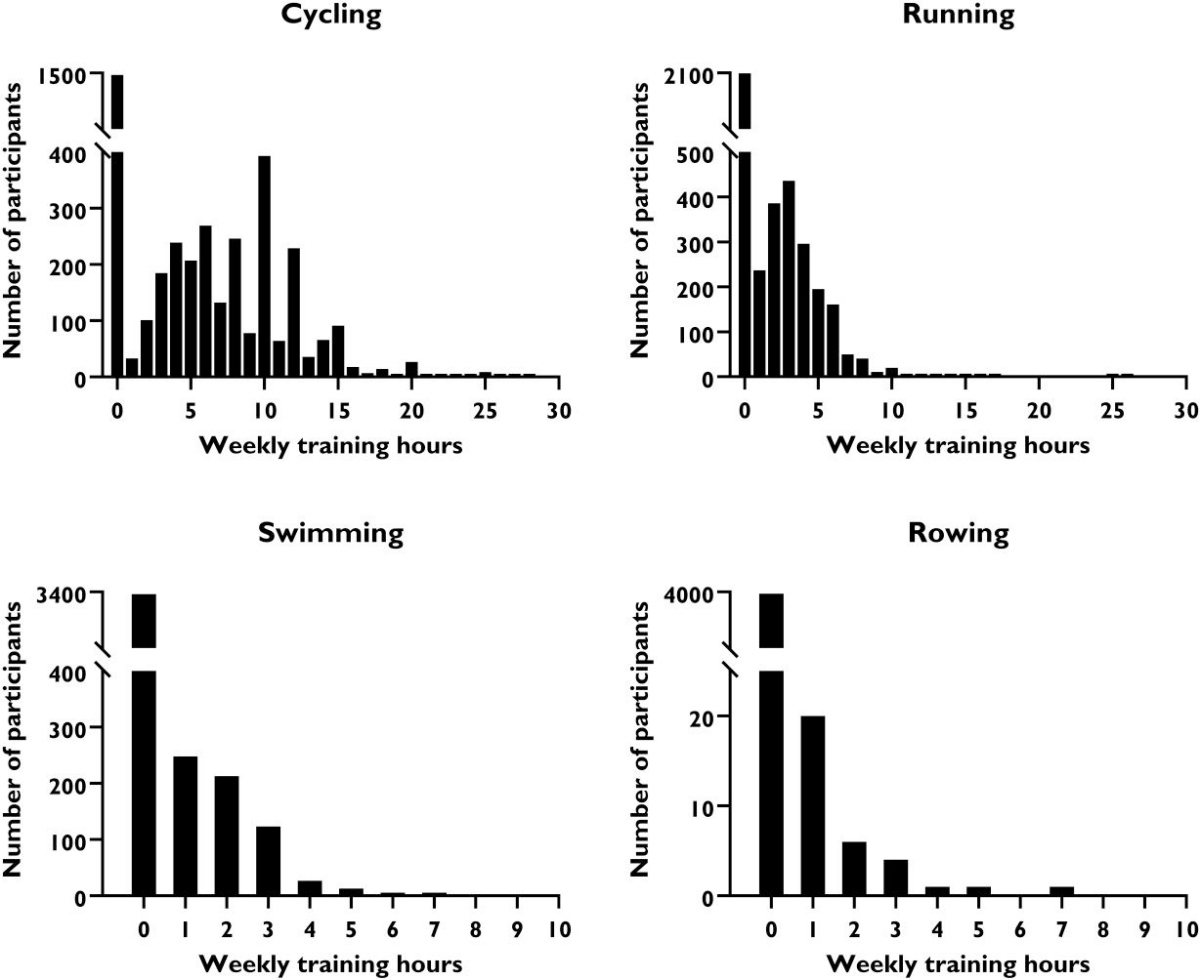
Distribution of weekly endurance exercise hours. Panel (*A*), (*B*), (*C*), and (*D*) show the distribution of exercise hours per week for cycling, running, swimming and rowing respectively of participants who filled in the screening questionnaire.

*Table 2* lists the population characteristics by self-reported weekly endurance exercise training. *Figure 1* shows the distribution of weekly exercise hours for each endurance sport category.

**Table 2:** Baseline characteristics by endurance exercise exposure

	Q1 (*n* = 985)	Q2 (*n* = 985)	Q3 (*n* = 984)	Q4 (*n* = 985)	*P* value
Age (years)	54 [49–61]^a,b^	54 [48–60]^a^	55 [49–61]^b^	57 [51–63]^c^	**<0**.**001**
Body height (cm)	178 [174–183]	179 [174–183]	179 [174–183]	178 [174–183]	0.313
Weight (kg)	81 [73–88]^a^	78 [72–85]^b^	76 [71–83]^b^	75 [70–81]^c^	**<0**.**001**
Alcohol consumption (units per week)	5 [2–8]^a^	5 [2–8]^a^	5 [2–8]^a^	4 [2–7]^a^	**0**.**048**
Current or past smoker—*n* (%)	379 (38.5)^a^	365 (37.1)^a^	334 (33.9)^a,b^	308 (31.3)^b^	**0**.**004**
Coronary artery disease—*n* (%)	48 (4.9)	44 (4.5)	35 (3.6)	44 (4.5)	0.536
Drugs					
Antidiabetic—*n* (%)	21 (2.1)	10 (1.0)	11 (1.1)	14 (1.4)	0.149
Antihypertensive—*n* (%)	103 (10.5)	89 (9.0)	103 (10.5)	79 (8.0)	0.181
Lipid-lowering—*n* (%)	151 (15.3)	138 (14.0)	131 (13.3)	147 (14.9)	0.579
History of AF/AFL—*n* (%)	47 (4.8)^a^	75 (7.6)^a,b^	79 (8.0)^b^	95 (9.6)^b^	**0**.**001**
Endurance exercise					
Lifetime exercise (hrs)	0 [0–863]^a^	3671 [2706–4701]^b^	8499 [7142–10 177]^c^	18 944 [15 097–24 398]^d^	**<0**.**001**
Weekly exercise (hrs)	0 [0–3]^a^	6 [3–8]^b^	8 [5–10]^c^	12 [10–14]^d^	**<0**.**001**
Cycling—*n* (%)	173(17.6)^a^	615 (62.4)^b^	748 (76.0)^c^	920 (93.4)^d^	**<0**.**001**
Running—*n* (%)	310 (31.5)^a^	560 (56.9)^b^	558 (56.7)^b^	416 (42.2)^c^	**<0**.**001**
Swimming—*n* (%)	39 (4.0)^a^	146 (14.8)^b^	201 (20.4)^c^	247 (25.1)^c^	**<0**.**001**
Rowing—*n* (%)	6 (0.6)	5 (0.5)	9 (0.9)	13 (1.3)	0.192
No Regular Endurance Exercise—*n* (%)	539 (54.7)^a^	0 (0)^b^	0 (0)^b^	0 (0)^b^	**<0**.**001**

Data presented as mean ± standard deviation, median [25th percentile—75th percentile] or number (%). Abbreviations—AF: atrial fibrillation, AFL: atrial flutter. Different letters (a, b, c, d) indicate significant pairwise differences (*P* < 0.05) with Bonferroni correction applied; same letters indicate no significant difference.

### Relationship between endurance exercise volume and AF/AFL

Unadjusted AF/AFL prevalence increased across lifetime exercise quartiles: 4.8% in Q1, 7.6% in Q2, 8.0% in Q3 and 9.6% in Q4 (*P* < 0.001), with Q3 and Q4 significantly higher compared to the bottom quartile.

In a logistic regression model adjusting for age, height, weight, smoking, alcohol consumption, and use of antidiabetic, antihypertensive, and lipid-lowering medication, lifetime exercise hours remained independently associated with AF/AFL prevalence. Compared with the lowest quartile, the odds ratio of AF/AFL was higher in Q2 (OR 1.86, 95% CI [1.26–2.73], *P* = 0.002), Q3 (OR 1.90, 95% CI [1.29–2.78], *P* = 0.001), and Q4 (OR 2.16, 95% CI [1.48–3.15], *P* < 0.001).

In an additional model with the same covariates, lifetime hours of endurance exercise (per 1000 h) remained independently associated with AF/AFL prevalence; analysed continuously, each additional 1000 h of lifetime endurance exercise was associated with a 2% higher odds ratio of AF/AFL (OR 1.02, 95% CI [1.01–1.03], *P* = 0.002).

Further exploratory analysis using penalized smoothing splines did not reveal a non-linear relationship between lifetime exercise hours and AF/AFL, and there was no significant risk reduction at lower lifetime exercise volumes.

**Figure 2:**
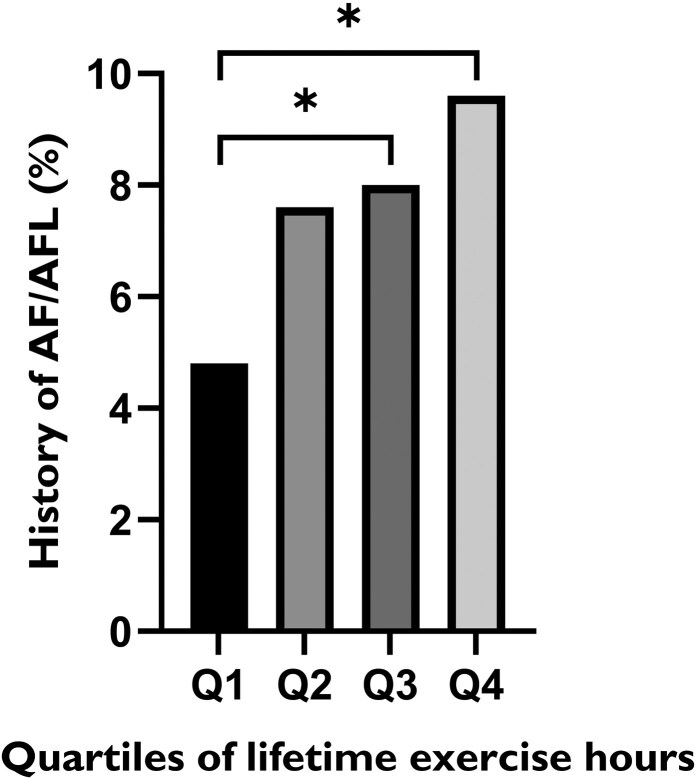
Prevalence of self-reported AF/AFL history by lifetime exercise hours quartile. Asterisk (*) indicates statistical significance based on chi-squared test with Bonferroni-adjusted post-hoc analysis.

**Figure 3:**
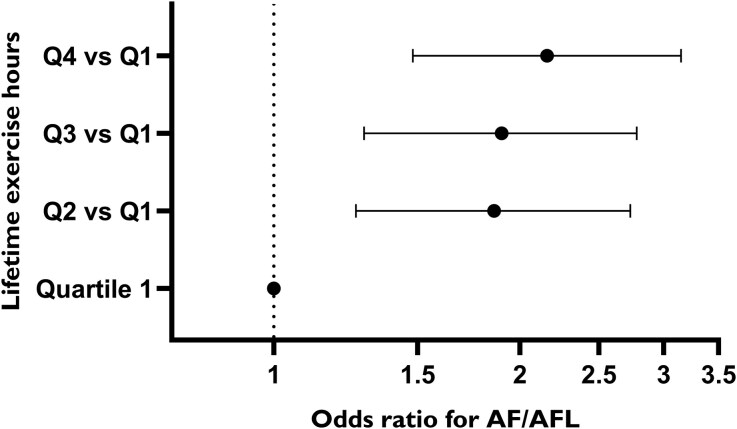
Relationship between lifetime exercise hours and AF/AFL. Odds ratios (OR) and 95% confidence intervals (CI) for atrial fibrillation by lifetime exercise hours quartile. ORs and 95% CIs are derived from a multivariable logistic regression model with Q1 as reference group and adjusted for age, body height, weight, smoking status, alcohol consumption, coronary artery disease, and use of antidiabetic, antihypertensive, and lipid-lowering medication. Abbreviations – AF: atrial fibrillation, AFL: atrial flutter.

*Figure 2* shows unadjusted AF/AFL prevalence across lifetime exercise quartiles. Adjusted odds ratios from multivariable logistic regression are presented in *Table 3* and *Figure 3*.

**Table 3:** Logistic regression analysis of AF/AFL according to quartiles of lifetime endurance exercise hours

	Univariable model		Multivariable model*	
	OR [95% CI]	*P* value	OR [95% CI]	*P* value
Quartile 1	—	—	—	—
Quartile 2	1.64 [1.13–2.40]	**0.009**	1.86 [1.26–2.73]	**0**.**002**
Quartile 3	1.74 [1.20–2.53]	**0.003**	1.90 [1.29–2.78]	**0**.**001**
Quartile 4	2.13 [1.48–3.06]	**<0.001**	2.16 [1.48–3.15]	**<0**.**001**

Quartile 1 as reference. OR: odds ratio, CI: confidence interval. * Multivariable model adjusted for age, body height, weight, smoking, alcohol consumption, coronary artery disease, and use of antidiabetic, antihypertensive, and lipid-lowering medication.

**Figure 4:**
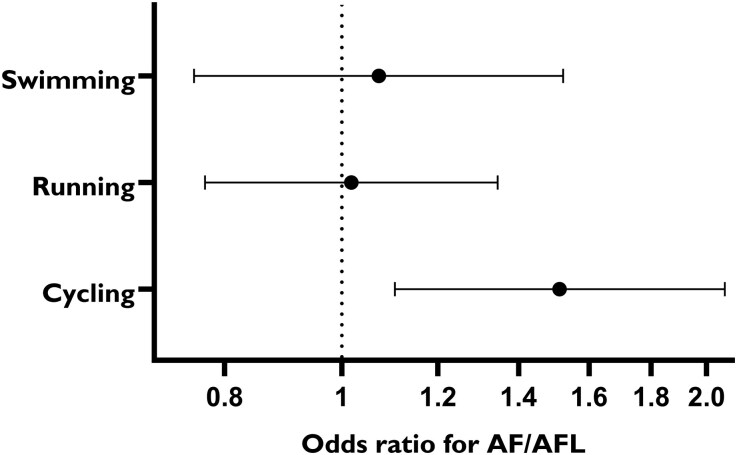
Relationship between sports discipline and atrial fibrillation/atrial flutter. Odds ratios (OR) and 95% confidence intervals (CI) for atrial fibrillation by sports discipline. ORs and 95% CIs are derived from a multivariable logistic regression model adjusted for lifetime exercise hours, age, body height, weight, smoking status, alcohol consumption, coronary artery disease, and use of antidiabetic, antihypertensive, and lipid-lowering medication. Abbreviations – AF: atrial fibrillation, AFL: atrial flutter.

In a multivariable logistic regression model including cycling, running, and swimming and adjusted for the same set of risk factors as well as total lifetime exercise hours, only cycling was significantly associated with a history of AF/AFL (adjusted OR 1.51 [1.11–2.07], *P* = 0.010). *Figure 4* shows adjusted odds ratios from multivariable logistic regression. A model including sports disciplines continuously (lifetime hours per sport) yielded similar results (see Supplementary File  *S2*).

A sensitivity analysis excluding participants reporting extreme endurance volumes (>20 h/week) yielded similar results (see Supplementary File  *S3*).

## Discussion

In this large cohort of middle-aged men, we observed a progressive increase in the prevalence of AF/AFL with higher volumes of lifetime endurance exercise. After adjusting for traditional risk factors, individuals in quartiles 2–4 (>1778 lifetime hours of exercise) had significantly higher odds ratios of reporting AF/AFL compared with the bottom quartile. Furthermore, our results indicate a further risk increase with even greater cumulative exercise exposure, with the largest effect seen in the 4th quartile. As a novel result, further analysis revealed a sport-specific pattern with cycling, but not running or swimming, being associated with increased AF/AFL risk. These findings suggest that both the volume and nature of the endurance activity, should be considered when evaluating arrhythmic risk in athletes.

### Impact of endurance exercise volume in a low-risk population

Our main finding is a positive association between AF/AFL history and lifetime exercise volume in this cohort of healthy middle-aged males. Participants with AF/AFL had greater lifetime endurance exposure and AF/AFL prevalence increased across quartiles of lifetime exercise hours. Although partly explained by older age in those with AF/AFL, there was also a trend towards higher weekly exercise hours in those with AF/AFL. Adjusted for traditional cardiovascular risk factors, quartiles 2 through 4 of lifetime exercise hours exhibited a higher likelihood of reporting a history of AF/AFL compared to the bottom quartile (≤1778 h). The top quartile (>12 231 h) showed a 2.1–fold higher likelihood of AF/AFL.

Consistent with current literature, our findings indicate that greater cumulative lifetime endurance exercise exposure is associated with increased AF/AFL risk.^5,12^ Prior case-control studies evaluating total lifetime exercise exposure have proposed similar potential thresholds for AF and AFL risk. Calvo et al. identified 2000 lifetime hours as a threshold above which AF risk increased, while Elosua et al. reported a comparable threshold around 1500 h.^2,13^ Consistently, the lower cut-off for Q2 in our cohort was 1778 lifetime hours, with Q2 showing a 1.8–fold higher likelihood of AF. Notably, whereas both prior studies suggested a plateau in AF occurrence beyond their proposed thresholds, AF/AFL risk in our cohort continued to increase with greater accumulated exercise exposure, reaching a 2.1-fold higher likelihood in Q4 (median 18 944 lifetime hours). This further increase in risk is likely a direct cause of the substantially broader exposure range captured in our population. Similarly, a prior Norwegian study found a progressive increase in both AF and AFL risk with increasing exercise exposure, with individuals accumulating approximately 1560 exercise hours over 20 years exhibiting a significantly higher prevalence of AF.^5^

While Calvo et al. reported a protective effect of lower exercise volumes (<2000 h) compared with truly sedentary individuals, neither our analysis nor that of Elosua et al demonstrated a significant risk reduction at lower cumulative exposure levels. In our cohort, this may reflect a degree of selection bias towards more active participants, despite efforts to include truly sedentary individuals.

Considering the above, it seems unlikely that the relationship between AF/AFL and cumulative exercise exposure is governed by a universal threshold but rather characterized by strong interindividual variation.^12^ Nevertheless, it appears that a certain cumulative training load is required to promote AF/AFL development. Volume (i.e. lifetime hours) may be more important than intensity in this regard, as our group recently demonstrated cardiac remodelling in athletes to be driven predominantly by training duration rather than intensity.^14^

One potential explanation for the lack of protective effect at moderate exercise amounts in our cohort, is the relatively low burden of cardiovascular risk factors in our cohort. For example, the ongoing CARDIA study reported a mean BMI of 29 kg/m^2^ and 9% diabetes prevalence, compared to 24 kg/m^2^ and 1% treated for diabetes in our cohort.^15^ As the protective effect of physical activity against AF is thought to be mediated through its beneficial impact on traditional cardiovascular risk factors, the relative absence of these factors in our cohort may have masked the initial protective slope of the J-curve, potentially revealing a more direct effect of exercise volume itself. As a second explanation, exercise may already convey cardiovascular benefits at relatively low, guideline-recommended levels of physical activity.^4,16^ The study questionnaire focused on structured endurance exercise and did not capture low-intensity physical activity such as brisk walking, commuting or occupational tasks. This highlights the importance of accurate and comprehensive assessment of physical activity in future prospective studies.

An important consideration is the observed prevalence of reported AF/AFL history in our cohort, ranging from 4.8% in the bottom quartile to 9.6% in the top quartile. Whilst these prevalences align with existing literature on AF in athletes—reporting between 0.3% and 28.5%^17–20^—the 4.8% prevalence observed even in the bottom quartile appears relatively high. These disparities are likely predominantly driven by age; e.g. Johansen et al. reported an AF prevalence of 28.6% among skiers with a median age of 68 years compared to 55 years in our cohort. Furthermore, a degree of recruitment bias is inherent to self-referred questionnaire data. Individuals with a history of atrial arrhythmia may have been more interested in the Master@Heart study in view of the study goals. Other potential explanations include residual effects of prior unreported exercise exposure, and lack of ECG confirmation (i.e. participants may have incorrectly labelled other types of palpitations or arrhythmias as ‘AF’ or ‘AFL’).

### Impact of endurance sport type on AF/AFL risk

Beyond cumulative exercise exposure, this cohort of cyclists, runners and swimmers offers new insights into the potential impact of these specific sports disciplines on AF/AFL risk. Given the high popularity of cycling in the Flemish region where the study was conducted, cyclists were particularly well represented. This demographic context enabled us to explore the potential influence of this sport type on AF/AFL risk in greater detail.

The phenotype and extent of cardiac adaptation vary across exercise modalities. Cycling in particular appears to be associated with profound remodelling, with greater left ventricular cavity dimensions compared with runners or rowers for example.^21,22^ Similarly, cycling has been identified as a major determinant of left atrial enlargement, although direct comparisons to other endurance sports are lacking.^17^ While greater average training volumes in cyclists, also evident in our cohort, may partly explain these differences, sport-specific mechanisms likely contribute. Cycling combines high dynamic and moderate static components, as opposed to running which is predominantly dynamic. Sustained isometric upper body strain during cycling has been hypothesized to increase cardiac pressure load, potentially augmenting cardiac remodelling.^23^

In our cohort of cyclists, runners, and swimmers, individuals with AF/AFL were more frequently cyclists and less frequently runners whereas swimmers were similarly represented. Notably, after adjustment for traditional risk factors and cumulative exercise volume, cycling remained independently associated with AF/AFL. Although cycling, running, and swimming have individually been associated with an increased risk of AF or AFL, direct comparisons between different sport types are rare.^20,24,25^ Several studies have demonstrated an increased AF prevalence among runners, including the landmark study by Karjalainen et al.^25–27^ Evidence among swimmers is limited, but one study reported a higher AF prevalence among swimmers with a median age of 73 years compared with an age-matched control group.^28^ Notably, Aizer et al. reported no increased AF risk among recreational cyclists. However, the mean weekly exercise exposure in that cohort (108 min) may have been insufficient to increase AF risk.^3^ In contrast, participants with AF in our study reported a median of 8 h of exercise per week.

Taken together, our findings suggest that among high-volume endurance athletes, cycling in particular is associated with AF/AFL independent of training volume, supporting that sport-specific loading characteristics may contribute to AF risk beyond cumulative exercise volume. While direct comparisons are lacking, many prior studies demonstrating an increased AF risk in athletes have focused on cohorts of cross-country skiers.^5,19,29,30^ In this context it is interesting to consider the parallels between cycling and skiing, as both disciplines combine prolonged endurance exercise with a moderate to high static upper-body component.^23,31^ Besides greater structural cardiac remodelling, this may confer a greater predisposition to atrial arrhythmias. Supporting this concept, a recent Australian study reported a particularly high prevalence of AF (28.5%) among former elite rowers, an endurance sport characterized by even greater upper-body involvement and static loading.^32^

Finally, while profound atrial structural remodelling likely plays a role, the pathophysiology of atrial arrhythmias in athletes is thought to involve autonomic modulation and systemic inflammation.^7–10^ Whether these factors differ between cyclists and other endurance sports remains to be studied.

### Limitations

This study has several limitations. The cross-sectional design and reliance on self-reported data limit causal inference and introduce the potential for different types of bias. Questionnaires did not capture exercise intensity or low-intensity physical activity such as walking or daily occupational tasks, which may underestimate total physical activity exposure. Importantly, cumulative lifetime hours of exercise were calculated rather than directly reported, extrapolating current weekly exercise hours across reported years of training, and thus assuming stable weekly training over time. AF/AFL history was entirely self-reported by a single questionnaire item without validation through ECG or medical records, posing a risk for misclassification and over- or underestimation of true AF/AFL prevalence. Similarly, risk factor assessment was based on reported medication use, which may not fully reflect underlying cardiovascular risk burden. Furthermore, the study included only male participants, and results may not be directly extrapolated to women. Additionally, the study lacks information on socioeconomic status of participants, which may have influenced AF prevalence. Lastly, a degree of recruitment bias cannot be excluded, as individuals with heightened health awareness or prior symptoms may have been more likely to participate in the screening for the Master@Heart study.

## Conclusion

In middle-aged males, greater self-reported estimated lifetime endurance exercise volume (>1778 lifetime hours) was associated with a higher odds ratio of self-reported AF or AFL, adjusting for age and traditional risk factors. The highest quartile (>12 231 lifetime hours) showed the greatest likelihood of AF or AFL. These findings contribute to growing evidence suggesting that high-volume endurance training may increase the risk of AF, even in individuals with a favourable cardiovascular risk profile. As a novel finding, specifically cycling appeared to be associated with greater AF risk, while this association was not found for running or swimming. Future studies should lay focus on accurate quantification of exercise exposure and sports type, combined with comprehensive structural and electrical phenotyping.

## Supplementary Material

oeag089_Supplementary_Data

## Data Availability

The data that support the findings of this study are available upon reasonable request.
