# Supplementary material for: Association of self-reported sports volume and discipline with atrial arrhythmia prevalence in middle-aged males
Source: Eur Heart J Open. 2026 May 22;6(3):oeag089. doi: 10.1093/ehjopen/oeag089 (PMC13310086; doi:10.1093/ehjopen/oeag089)
Supplement: oeag089_Supplementary_Data [file oeag089_supplementary_data.zip › Supplementary File 1.docx]

**Supplementary File 1 – Master@Heart screening questionnaire**

The following questionnaire was made using Redcap in Dutch. The questionnaire is accessible via: <https://redcap.gbiomed.kuleuven.be/surveys/?s=FJX8DY74TA>

The link is only available after you have completed a registration on the website and received a registration code.

Redcap makes use of several question options, for example an open field or drop down menu with options available. Behind the question, in brackets, the used option is depicted and the options are displayed. Some questions are branched, which means they are only visible when another question is answered. If, for example, you never smoked, you will not see other questions involving past or present smoking behaviour.

Please fill in the following questionnaire by truth.

Firstly you will need to provide your registration code. Please make sure to use capitals when necessary, afterwards the next questions will appear.

Thank you in advance.

Questionnaire

1. Provide your registration code, make sure to use capitals when appropriate. (Open field, if the code corresponds to the database the next questions will appear)
2. I agree that the provided data can be used confidentially for scientific research. The data can only be used in the context of this study. The data will be coded so that they cannot directly be linked to your identity. (Drop down, Agree or not agree)
3. I agree to be contacted to participate in the research project, which includes several examinations for the heart. These include a coronary CT, a cardiac MR, if I get selected. This is voluntary and without any commitment. (Drop down, Agree or not agree)

Demography

1. Sex (Drop down, male or female)
2. Birthday (Date field)
3. Date of registration (Date field)
4. Highest education (Drop down, primary school; high school; university; I still go to school)
5. Do you work? (Yes or no)
   1. Do you work with fixed hours? (Drop down, yes; no I work in shifts; I work in shifts including night shifts; I’m self-employed)
   2. How many hours do you work per week? (Drop down, 1;2;…,>42)
6. What is the name of your personal physician? (Open field)
7. What is your length in cm? (Open field)
8. What is your weight in kg? (Open field)
9. Do you smoke? (Drop down, I never smoked; I quit smoking; I still smoke)
   1. If you quitted smoking.
      1. How long ago did you stop smoking? (Drop down, less than 5 years; less than 10 years; more than 10 years; more than 20 years)
      2. How long did you smoke? (Drop down, less than 1 year; less than 2 years; less than 3 years; less than 5years; less than 10 years; more than 10 years)
   2. If you still smoke
      1. How long do you smoke? (drop down, less than 1 year; less than 2 years; less than 3 years; less than 5years; less than 10 years; more than 10 years)
10. Do you take medication for diabetes? (Yes or no)
11. How many alcoholic beverages do you drink per week? (Drop down, I drink no alcohol; 1; 2; 3; 4;…; >14)

Current sport activity

If you are currently obtaining a bachelor or master in kinesiology, the sport lessons do not count as weekly sport activity. Unless you are studying at the sport schools and are performing part of your trainings during school hours.

1. Do you, at this moment, participate in weakly sport activities? (Yes or no)
   1. If yes, which sports do you do weekly? (Multiple answers possible)
      1. Cycling
      2. Running (>1500m)
      3. Running (<1500m)
      4. Triathlon (If selected questions for swimming, running and cycling will be displayed)
      5. Swimming
      6. Football
      7. Basketball
      8. Handball
      9. Golf
      10. Chess
      11. Dancing
      12. Gymnastics
      13. Omni sport
      14. Rowing
      15. Darts
      16. Weight lifting, powerlifting
      17. Badminton
      18. Tennis
      19. Duathlon (If selected questions for running and cycling will be displayed)
      20. Fighting sports
      21. Other (specify this sport in an open field)
      22. Volleyball
   2. For every sport selected above the following questions will appear:
      1. At which age did you start (selected sport)? (Drop down, 1; 2;…; 69)
      2. Did you ever stop (selected sport) longer than 3 years? (Yes or no)
      3. Do you participate in (selected sport) for over half a year? (Yes or no)
      4. At which level do you participate in (selected sport)? (Drop down, recreationally; recreational competition, competition regionally; competition nationally; competition international)
      5. How many hours do you train for the (selected sport)? (Drop down, 1; 2;…; >30)
2. Do you perform strength training in a fitness centre? (Yes or no)
   1. If yes, how many strength training do you perform
      1. 1 time per week
      2. 2 times per week
      3. 3 times per week
      4. 4 times per week
      5. 5 times per week
      6. 6 times per week
      7. Daily
3. If you work, how do you go to work?
   1. Public transport
   2. Car
   3. Bike
   4. Running
   5. Walking
   6. Cycling or running with car or public transport
      1. If you cycle or run, how long do you run or cycle to work each week?
         1. <1hour
         2. >1hour
         3. >2hours
         4. >3hours
         5. >4hours
         6. >5hours
         7. >6hours
         8. >7hours
         9. >8hours
         10. >9hours
         11. >10hours
4. If you still go to school, how do you go to school?
   1. Public transport
   2. Car
   3. Bike
   4. Running
   5. Walking
   6. Cycling or running with car or public transport
      1. How long do you run or cycle to school each week?
         1. <1hour
         2. >1hour
         3. >2hours
         4. >3hours
         5. >4hours
         6. >5hours
         7. >6hours
         8. >7hours
         9. >8hours
         10. >9hours
         11. >10hours

Past sport activity

If you in the past followed an education, which included sport lessons as an important part of your education, please do not count this as sport in the past, unless this was sport for training purposes in a sport school.

1. Which sports did you perform in the past?
   1. Cycling
   2. Running (>1500m)
   3. Running (<1500m)
   4. Triathlon (If selected questions for swimming, running and cycling will be displayed)
   5. Swimming
   6. Football
   7. Basketball
   8. Handball
   9. Golf
   10. Chess
   11. Dancing
   12. Gymnastics
   13. Omni sport
   14. Rowing
   15. Darts
   16. Fitness
   17. Weight lifting, powerlifting
   18. Badminton
   19. Tennis
   20. Duathlon (If selected questions for running and cycling will be displayed)
   21. Fighting sports
   22. Other (specify this sport in an open field)
   23. Volleyball
   24. I never participated in sports
       1. For every sport selected above the following questions will appear:
          1. At which age did you start (selected sport)? (Drop down, 1; 2; 3;…; 70)
          2. At which age did you stop (selected sport)? (Drop down, 1; 2; 3;…; 70)
          3. At which level did you participate at this sport? (Drop down, recreationally; recreational competition, competition regionally; competition nationally; competition international)
          4. How many hours did you train per week for (selected sport)? (Drop down, 1; 2; 3; …; 30)
       2. If fitness, how many times per week did you go to a fitness centre for strength training. (Drop down, 1; 2; 3; 4; 5; 6; 7)

Health questions

1. Have you ever been examined or treated for chest pain or breathlessness? (Yes or no)
2. Have you ever been examined or treated for chest pain or breathlessness during exercises? (Yes or no)
3. Have you ever been examined or treated for palpitations or cardiac arrhythmias? (Yes or no)
   1. If yes, did this involve atrial fibrillation or atrial flutter? (Yes or no)
   2. Did you receive a pacemaker or defibrillator? (Yes or no)
4. Have you ever been examined or treated for dizziness during or after exercise? (Yes or no)
5. Have you ever fainted during or after exercise? (Yes or no)
6. Has a doctor ever mentioned you have a heart murmur? (Yes or no)
7. Has a doctor ever mentioned you have an elevated blood pressure? (Yes or no)
8. Do you take medication for an elevated blood pressure? (Yes or no)
9. Has a doctor ever mentioned you have high cholesterol levels? (Yes or no)
10. Do you take medication for high cholesterol levels? (Yes or no)
11. Are you known to have problems of the coronary arteries? (Yes or no)
12. Do you have other complains of the heart or blood vessels? (Yes or no)
    1. If yes, which? (Open field)
13. Do you take medication at this moment?
    1. If yes, which? (Only mention the name; open field)
14. Have you even been diagnosed with asthma? (Yes or no)
15. Have you ever suffered from coughing, shortness of breath or breathing disorders during or after exercise? (Yes or no)
16. Do you use or have you used inhalation medication? (Yes or no)
17. Do you have any allergies (pollen, medication, food, insects)? (Yes or no)
    1. If yes, which? (Open field)
18. Other lung problems? (Yes or no)
    1. If yes, which? (Open field)

Family disorders

1. Has a stroke or myocardial infarction before the age of 65y occurred in a family member? (Yes or no)
2. Has sudden death before the age of 50y occurred in a family member? (Yes or no)

Participation in the study

1. In which testing centre would like to undergo testing? You can provide multiple answers. (Drop down, UZ Leuven; UZ Antwerpen; Jessa Hospital Hasselt)
2. Do you suffer from claustrophobia?

Please push “submit” if you have answered all questions. If you did not see, any other questions appear after your registration code, you probably provided the wrong code. Please check your code, which is capital sensitive.
