# Supplementary material for: Association of self-reported sports volume and discipline with atrial arrhythmia prevalence in middle-aged males
Source: Eur Heart J Open. 2026 May 22;6(3):oeag089. doi: 10.1093/ehjopen/oeag089 (PMC13310086; doi:10.1093/ehjopen/oeag089)
Supplement: oeag089_Supplementary_Data [file oeag089_supplementary_data.zip › Supplementary File 2_Revised2.docx]

**Supplementary File 2 – Multivariable logistic regression for AF by sport discipline**

|  | Univariable model | | Multivariable model* | |
| --- | --- | --- | --- | --- |
|  | OR [95% CI] | P value | OR [95% CI] | P value |
| **AF Risk vs. Lifetime Exercise Hours per Sports** | | | | |
| Cycling  (per 1000 hrs) | 1.022 [1.010 – 1.035] | **<0.001** | 1.018 [1.005 – 1.031] | **0.008** |
| Running  (per 1000 hrs) | 1.016 [0.989 – 1.043] | 0.253 | 1.027 [1.000 – 1.054] | 0.052 |
| Swimming  (per 1000 hrs) | 1.036 [0.948 – 1.133] | 0.431 | 1.033 [0.944 – 1.130] | 0.481 |
| **AF Risk vs. Binary Sports Exposure** | | | | |
| Cycling | 1.57 [1.21 – 2.04] | **<0.001** | 1.51 [1.11 – 2.07] | **0.010** |
| Running | 0.74 [0.58 – 0.94] | **0.013** | 1.02 [0.77 – 1.35] | 0.898 |
| Swimming | 1.01 [0.73 – 1.40] | 0.943 | 1.07 [0.76 – 1.52] | 0.695 |

**Supplementary File 2 – Relationship between sports type and atrial fibrillation (AF).**OR: odds ratio, CI: confidence interval. * Multivariable model adjusted for age, body height, weight, smoking, alcohol consumption, coronary artery disease, and use of antidiabetic, antihypertensive, and lipid-lowering medication. Model for binary sports exposure additionally adjusted for total lifetime exercise hours. Participants were considered active in a given sport for binary analysis if they reported ≥1 hour per week.
