# Supplementary material for: Association of self-reported sports volume and discipline with atrial arrhythmia prevalence in middle-aged males
Source: Eur Heart J Open. 2026 May 22;6(3):oeag089. doi: 10.1093/ehjopen/oeag089 (PMC13310086; doi:10.1093/ehjopen/oeag089)
Supplement: oeag089_Supplementary_Data [file oeag089_supplementary_data.zip › Supplementary File 3_Revised2.docx]

**Supplementary File 3 – Sensitivity analysis of the association between exercise and AF after excluding extreme exercise values (>20 hrs/week)**

| **Lifetime exercise quartile** | **Lifetime exercise hours** | **History of AF** | **OR [95% CI]** | **P value** |
| --- | --- | --- | --- | --- |
| **Q1 (n = 985)** | 0 [0 – 863] | 47 (4.8) | - | - |
| **Q2 (n = 983)** | 3671 [2709 – 4697] | 75 (7.6) | 1.84 [1.25 – 2.70] | **0.002** |
| **Q3 (n = 983)** | 8499 [7141 – 10172] | 79 (8.0) | 1.85 [1.26 – 2.71] | **0.002** |
| **Q4 (n = 945)** | 18496 [14953 – 23986] | 92 (9.7) | 2.13 [1.46 – 3.10] | **<0.001** |

**Multivariable logistic regression for lifetime exercise hours and AF**OR: odds ratio, CI: confidence interval. Training hours reported as median [25th – 75th percentile]. History of AF reported as N (%). Multivariable model adjusted for age, body weight, height, smoking, alcohol consumption, coronary artery disease, and use of antidiabetic, antihypertensive, and lipid-lowering medication.

|  | **Odds Ratio [95% CI]** | **P value** |
| --- | --- | --- |
| **AF Risk vs. Lifetime Exercise Hours per Sports** | | |
| Cycling (per 1000 hrs) | 1.018 [1.005 – 1.033] | **0.009** |
| Running (per 1000 hrs) | 1.020 [0.985 – 1.057] | 0.266 |
| Swimming (per 1000 hrs) | 1.048 [0.957 – 1.147] | 0.312 |
| **AF Risk vs. Binary Sports Exposure** | | |
| Cycling | 1.54 [1.12 – 2.12] | **0.008** |
| Running | 1.01 [0.76 – 1.33] | 0.945 |
| Swimming | 1.12 [0.79 – 1.59] | 0.530 |

**Multivariable logistic regression for AF by sport discipline**OR: odds ratio, CI: confidence interval. * Multivariable model adjusted for age, body weight, height, smoking, alcohol consumption, coronary artery disease, and use of antidiabetic, antihypertensive, and lipid-lowering medication. Model for binary sports exposure additionally adjusted for total lifetime exercise hours. Participants were considered active in a given sport for binary analysis if they reported ≥1 hour per week.
